# Pre-Pregnancy Diet Quality Is Associated with Lowering the Risk of Offspring Obesity and Underweight: Finding from a Prospective Cohort Study

**DOI:** 10.3390/nu13041044

**Published:** 2021-03-24

**Authors:** Dereje G. Gete, Michael Waller, Gita D. Mishra

**Affiliations:** Centre for Longitudinal and Life Course Research, School of Public Health, Faculty of Medicine, University of Queensland, 266 Herston Road, Brisbane, QLD 4006, Australia; m.waller@uq.edu.au (M.W.); g.mishra@uq.edu.au (G.D.M.)

**Keywords:** preconception, healthy eating index-2015, diet quality, offspring, obesity, underweight

## Abstract

Maternal diet plays a critical role in epigenetic changes and the establishment of the gut microbiome in the fetus, which has been associated with weight outcomes in offspring. This study examined the association between maternal diet quality before pregnancy and childhood body mass index (BMI) in offspring. There were 1936 mothers with 3391 children included from the Australian Longitudinal Study on Women’s Health (ALSWH) and the Mothers and their Children’s Health (MatCH) study. Maternal dietary intakes were assessed using a semi-quantitative and validated 101-item food-frequency questionnaire (FFQ). The healthy eating index (HEI-2015) score was used to explore preconception diet quality. Childhood BMI was categorized as underweight, normal, overweight, and obese based on sex and age-specific BMI classifications for children. Multinomial logistic regression with cluster-robust standard errors was used for analyses. Greater adherence to maternal diet quality before pregnancy was associated with a lower risk of offspring being underweight after adjustment for potential confounders, highest vs. lowest quartile (relative risk ratio (RRR) = 0.68, 95% confidence interval (CI): 0.49, 0.96). Higher adherence to preconception diet quality was also inversely linked with the risk of childhood obesity (RRR = 0.49, 95% CI: 0.24, 0.98). This association was, however, no longer significant after adjusting for pre-pregnancy BMI. Sodium intake was significantly associated with decreased risk of childhood overweight and obesity (RRR = 0.18, 95% CI: 0.14, 0.23) and (RRR = 0.21, 95% CI: 0.17, 0.26), respectively. No significant association was detected between preconception diet quality and offspring being overweight. This study suggests that better adherence to maternal diet quality before pregnancy is associated with a reduced risk of childhood underweight and obesity.

## 1. Introduction 

Childhood underweight, overweight, and obesity are increasingly rife, which have a substantial impact on adulthood health and quality of life. Globally, over 340 million children and adolescents aged 5–19 are overweight/obese [1]. In 2018, ∼8% of Australian children aged 5–14 were obese and 12% had overweight [2]. Childhood underweight, overweight, and obesity are linked with adverse health outcomes throughout the life-span [3]. For example, overweight/obese children are more likely to become obese adults [4] and have a greater risk of metabolic, pulmonary, and cardiovascular diseases, including diabetes, hypertension, stroke, and asthma. Childhood obesity diminishes social and emotional functioning, including depression, bullying, and low self-esteem [5,6]. Childhood underweight is also a serious public health concern, especially in low and middle-income countries, which has been linked with a greater risk of infectious diseases and leads to overweight and obesity in adulthood [7,8].

Maternal nutrition could affect offspring body composition or weight outcomes through epigenetic changes and the establishment of the gut microbiome in the fetus [9]. The maternal diet is also the key source of energy for the baby, which has been associated with the risk of offspring obesity [10,11]. Over the past decade, researchers have provided pertinent information on the relationship between maternal diets in pregnancy and offspring body compositions. Currently, however, researchers have shown a growing interest in examining the impacts of maternal diet before pregnancy on offspring body mass index (BMI) outcomes since the preconception diet plays a critical role in successful pregnancy outcomes and child health. The baby is fully formed by the end of the 12th week of pregnancy, as well as fetal tissue growth and developments are being programmed [12]. A pre-pregnancy diet has, therefore, a significant role in the successful development of a pregnancy and is likely to have a substantial impact on offspring outcomes. A study conducted by Stephenson et al. showed that nutritional intervention in pregnancy might ameliorate maternal health with a modest effect on adverse birth outcomes, but no latter impact on offspring health [13]. Maternal nutrition in pregnancy relies on the availability of micronutrient reserves, that is, stores of certain nutrients before pregnancy, including iron and calcium. Keeping adequate preconception nutrition, therefore, has a critical role in ensuring a good nutritional status in the pregnancy period and child health [14].

A considerable amount of literature has been published on the relationship between maternal diet in pregnancy and childhood body compositions or BMI outcomes, including overweight and obesity; however, the findings have been inconsistent [15,16,17,18]. A prospective cohort study conducted by Strohmaier et al. reported no association between peri-conception healthy dietary patterns and obesity risk in their offspring [19]. No study, to our knowledge, has conducted the relationship between pre-pregnancy diet quality and a child’s BMI outcomes in offspring.

The current study set out to examine the association between pre-pregnancy diet quality and childhood BMI outcomes aged 2–12 years using data from a nationally representative longitudinal study of Australian mothers and children. We hypothesized that maternal diet quality before pregnancy would be associated with a decreased risk of childhood underweight and overweight/obesity. The study also investigated the interaction effects of pre-pregnancy BMI in the relationship between preconception diet quality and offspring BMI.

## 2. Materials and Methods 

### 2.1. Study Design and Populations

This study utilized data from the Australian Longitudinal Study on Women’s Health (ALSWH) and Mothers’ and their Children’s Health (MatCH) study. The ALSWH is an ongoing, population-based prospective cohort study of Australian women that was initiated in 1996 to investigate factors affecting women’s health and well-being, and over 14,000 women born in 1973-1978 (18–23 years) were recruited. The study participants were randomly selected from the National Universal Health Insurance database (Medicare), which includes all Australian citizens and permanent residents, and the maternal information was collected at 3-year intervals from 1996 to 2015. A detailed description of the ALSWH has been published elsewhere [20].

In 2016/2017, 8929 ALSWH participants (1973–1978 cohort) were invited to be part of the MatCH sub-study, who had consented to be contacted about sub-studies. Overall, 3048 mothers provided complete information about their children born between 2003 and 2015 (*n* = 5799) [21].

**Figure 1:**
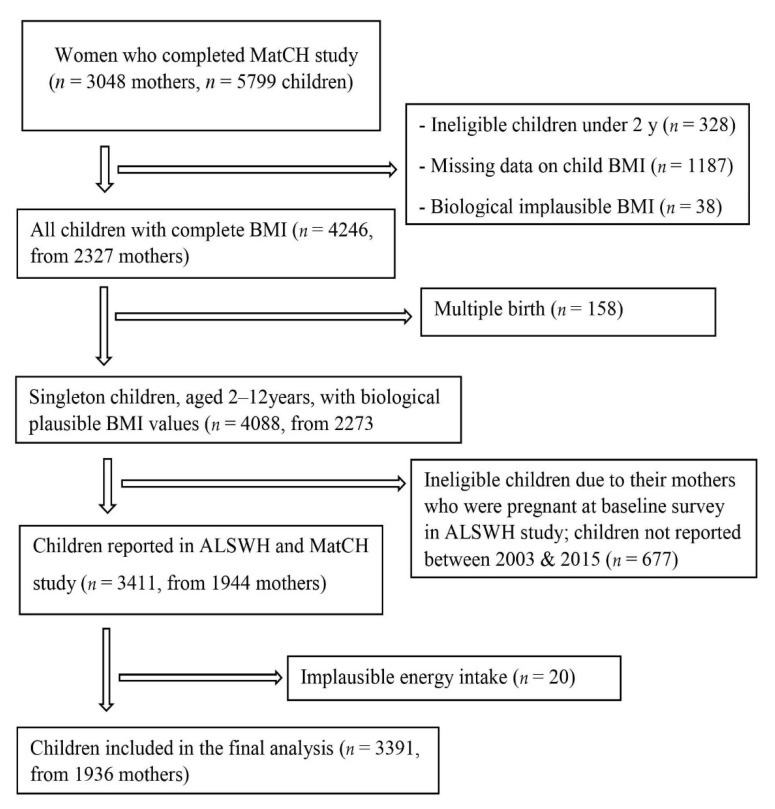
Flow diagram of the study sample from the Australian Longitudinal Study on Women’s Health (ALSWH) and Mothers and their Children’s Health (MatCH) study for the analysis of relationship between preconception dietary patterns and childhood body mass index (BMI).

In the present study, mothers who were non-pregnant and nulliparous at baseline Survey 3 or 5, and reported ≥ 1 live birth between 2003 and 2015 were included. Women who had implausible energy intake (>16,800 KJ/day or <2100 KJ/day) [22], had missing data on offspring obesity, multiple births, and biological implausible BMI [23] were excluded. All singleton children, born between Survey 3 and 7 and who were aged 2–12 years were included. A total of 3391 children, from 1936 mothers were included in the analyses (Figure 1). 12 years of follow-up data were included. The Human Research Ethics Committees at the University of Newcastle and the University of Queensland approved the ALSWH study, and all study subjects received informed consent. 

### 2.2. Dietary Assessment

Women’s dietary information was initially collected at Survey 3 (2003) and the follow-up phase, at Survey 5 (2009) using a semi-quantitative and validated 101-item food frequency questionnaire (FFQ) asking about a women’s habitual dietary intake over the previous one year. Daily intake of foods (in grams per day) and nutrients were estimated from the national government food composition database of Australian foods, the NUTTAB95 [24]. Women’s dietary consumption was assessed using the Dietary Questionnaire Epidemiologic Study version 2. The evaluation and development of FFQ have been described elsewhere [25]. The FFQ was validated for 63 women of child-bearing age against 7-days weighed food records who participated in an iron deficiency study [26]. 

This study used the healthy eating index (HEI-2015) score to assess preconception diet quality. The HEI-2015 score is a contemporary diet quality index, which consists of 13 dietary components that sum to a total maximum score of 100 points. Each of the dietary components is calculated based on a density of 1000 kcal [27,28]. 9 dietary components; total fruits, total vegetables, whole fruits, whole grains, greens and beans, total proteins, dairy, seafood/plant proteins, and fatty acids, are assessed for adequate consumption. Mothers with higher consumption get greater scores. However, 4 dietary components, including saturated fats, added sugars, refined grains, and sodium were to be consumed in moderation, in which mothers with lower consumptions get higher scores. Consumptions of foods (in cup or ounce per 1000 Kcal) were computed. The 6 dietary components (total fruits, total vegetables, whole fruits, whole grains, greens and beans, total protein, and seafood and plant proteins) are worth 0 to 5 points each, and the remaining 7 components are worth 0 to 10 points. A maximum point of HEI-2015 score is 100 indicating perfect adherence. The HEI-2015 score was further classified into quartiles according to the distribution of the study population. The HEI-2015 score was classified as quartile 1, low adherence (22.44–49.63), quartile 2, mild adherence (49.63–59.76), quartile 3, moderate adherence (59.77–67.87), and quartile 4, high adherence (67.88–86.93) for regression analyses and ease of interpretability. 

### 2.3. Assessment of Offspring BMI 

In the MatCH study, mothers were provided with measuring tapes with instructions to measure their offspring’s height. The study used the maternal self-report of their children’s weight and height to compute childhood BMI, which was computed as the weight in kilograms divided by the height in meters squared (kg/m^2^). Children over 2 years of age were categorized as underweight, normal, overweight, and obese according to sex and age-specific BMI classifications for children [29].

### 2.4. Assessment of Confounders and Covariates 

The present study assessed the dataset for potential confounders and covariates because of their known association with offspring BMI outcomes from previous literature. Information regarding maternal education, marital status, household income, alcohol intake, smoking status, pre-pregnancy BMI, total energy intake (TEI), and physical activity was controlled for using the maternal self-reported survey before the index birth (baseline Survey 3 or 5). Information on a hypertensive disorder in pregnancy (HDP) and gestational diabetes mellitus (GDM) were controlled for using the same survey which notified us of the index birth from Survey 4 to 7. 

The women’s age at birth (years) was computed using the women’s date of delivery and date of birth and treated as a continuous variable. The residence was classified into urban and rural/remote areas [30]. The alcohol consumption was classified as a non-drinker, low-risk drinker (≤14 drinks/week), risky drinker (15–28 drinks/week), and high-risk drinker (>28 drinks/week) based on the classifications of the National Health and Medical Research Council (NHMRC) in Australia [31]. Only eight women were high-risk drinkers (0.41%), so this was merged with the risky drinker group. Physical activity was derived from total metabolic equivalent [MET] values based on frequency and duration of walking and moderate and vigorous-intensity activity and classified as sedentary/low (<600 MET min/week), moderate (600 to 1200 MET min/week), or high (≥1200 MET min/week) [32]. The pre-pregnancy BMI was categorized as underweight (BMI < 18.5 kg/m^2^), normal weight (BMI 18.5 to < 25 kg/m^2^), overweight (25–30 kg/m^2^), and obese (≥30 kg/m^2^). However, we combined the underweight and normal-weight groups into healthy weight (BMI < 25 kg/m^2^) because there were a few women categorized as underweight (n = 66, 3.4%).

With regard to childhood factors in the offspring, women were also requested to provide their children’s age, sex, history of preterm birth (live birth ≤ 36 weeks of pregnancy), and low birth weight (LBW) (live birth weight < 2.5 kg), breastfeeding status, and dietary intake in the MatCH study. The child’s dietary consumption was classified as fruits and vegetables, fat from dairy, sweetened beverages, and non-core foods (high fat and sugar foods) [33]. The offspring dietary consumption either in the past 24 hours or in the previous week was explored using a validated 28-item children’s dietary questionnaire (CDQ) at all ages except for infants who have not commenced solid diets. 

### 2.5. Statistical Analyses

Statistical analysis was performed using Stata software version 16 (StataCorp) and SAS software version 9.4 (SAS Institute Inc., Cary, NC, USA). Analysis of variance (ANOVA) and Pearson’s chi-square were used to describe maternal and childhood characteristics according to the HEI-2015 score and offspring BMI categories. The study presented the descriptive statistics as percentages in each group (%) for categorical variables and means ± SDs for continuous variables. A multinomial logistic regression model was used to examine the relationship between pre-pregnancy diet quality and childhood BMI categories. A relative risk ratio (RRR) with a 95% confidence interval (CI) was estimated to evaluate the risk of offspring being underweight, overweight, and obese. The study used cluster-robust standard errors to account for correlations among siblings. This study also examined the relationship between each dietary component of HEI-2015 scores measured as continuous variables and offspring BMI categories. Several important potential confounders and covariates were adjusted. The first model was unadjusted; the second model adjusted for women’s socio-demographic and lifestyle factors (maternal education, smoking, physical activity, and household income); the third model further adjusted for childhood factors (offspring age, sex, diets, and breastfeeding); the final model further adjusted for pre-pregnancy BMI. The analysis was further stratified by Pre-pregnancy BMI categories since the pre-pregnancy BMI was previously reported as a strong predictor of adverse weight outcomes in offspring [34]. Sensitivity analysis was performed to examine the changes in the maternal HEI-2015 score from before to during pregnancy. Spearman’s correlation coefficient and paired t-test were conducted to evaluate the stability and changes of the HEI-2015 score at the two-time points. The study excluded missing data on the outcome variable and analyzed it as a list-wise deletion or complete case. However, we didn’t exclude or impute the missing data on potential confounders since they counted below 5% of the total sample size. *p*-value ≤ 0.05 was considered statistically significant. 

## 3. Results 

This study included 1936 mothers with 3391 children (mean age 7.4 years, SD 2.9) using the ALSWH and the MatCH study (Figure 1). Of the 3391 children, 391 (11.5%) were categorized as underweight, 424 (12.5%) as overweight, and 111(3.3%) as obese. The median period between the maternal FFQ survey and childbirth was 3.4 (IQR 2.9). The mean age of mothers at birth was 33.1 (SD 2.9) years.

**Figure 2:**
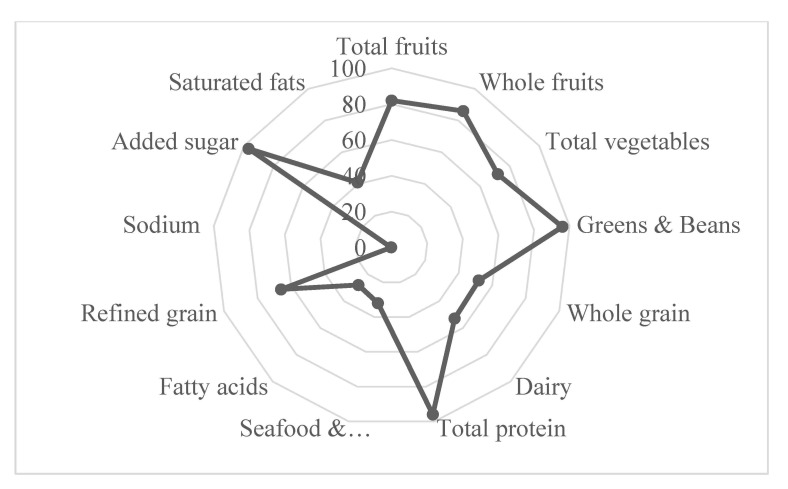
Spider plot indicating the percentage for each dietary component of HEI-2015 score before pregnancy (*n* = 1936).

As shown in Figure 2, women had good adherence to fruits, added sugar, total protein, and greens and beans, while they had low adherence to sodium consumption, saturated fats, fatty acids, and seafood and plant protein. The mean pre-pregnancy HEI-2015 score was 58.1 (SD 12.3). The HEI-2015 score had a strong inverse correlation with glycemic index (*r* = −0.5), glycemic load (*r* = −0.3), all fats (*r* = −0.4), and saturated fats (−0.5). 

A significantly higher proportion of childhood obesity was found among mothers with the lowest income, education, and obesity (Table 1). Underweight children are also more likely to be born to mothers with the lowest income and smokers. LBW children are more likely to be underweight and obese, compared to the normal BMI category. There was also a higher percentage of offspring obesity and underweight among children raised without breastfeeding. Childhood obesity was more likely to occur among children with high consumption of sweetened beverages. 

**Figure 3:**
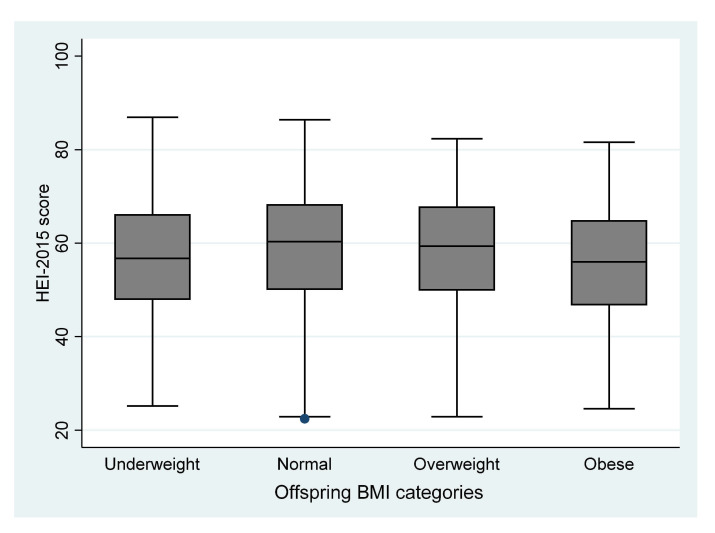
Distribution of pre-pregnancy HEI-2015 score over offspring BMI categories (*n* = 3391).

Figure 3 compares the distribution of HEI-2015 score before pregnancy over offspring BMI categories. The mean HEI-2015 score was lowest among obese (55.1 points) and underweight (56.7 points) children.

As can be seen from Table 2, there was a greater adherence to diet quality among older, urban residents, and well-educated women. Women with better adherence to maternal diet quality had also greater household income and performed higher physical activity.

Table 3 provides the relationship between pre-pregnancy diet quality and offspring BMI categories. In all models, women with the lowest quartile of HEI-2015 score and children with normal weight are used as reference groups. We found children of mothers with a greater quality of diet had a reduced risk of underweight and obesity compared to children of mothers with lower diet quality after adjustments for important potential confounders and covariates. Greater adherence to pre-pregnancy HEI-2015 score was associated with decreased the risk of childhood underweight, highest vs. lowest quartile (RRR = 0.68, 95% CI: 0.49, 0.96), *p* = 0.03. Maternal diet quality was also inversely associated with the risk of offspring obesity. Compared with Quartile 1, women in Quartile 4 of the HEI-2015 score had a lower risk of childhood obesity after adjusting for potential confounders, including maternal education, smoking status, physical activity, household income, child diets, sex, age, and breastfeeding status (RRR = 0.49, 95% CI: 0.24, 0.98), *p* = 0.04. However, the association was not remained significant after adjusting for pre-pregnancy BMI (RRR = 0.54, 95% CI: 0.26, 1.11), *p* = 0.09.

This study also assessed the relationship between each dietary component of HEI-2015 scores before pregnancy and childhood BMI categories (Table 4). Better adherence to sodium consumption was strongly associated with reduced risk of childhood overweight and obesity after fully adjusting for maternal and child characteristics (RRR = 0.18, 95% CI: 0.14, 0.23) and (RRR = 0.21, 95% CI: 0.17, 0.26), respectively, *p* < 0.0001. Higher adherence to seafood and plant proteins component was also inversely associated with risk of childhood underweight (RRR = 0.84, 95% CI: 0.73, 0.99), *p* = 0.03.

The study examined whether the association between preconception HEI-2015 score and childhood BMI categories differed by pre-pregnancy BMI using an interaction model (Supplemental Table S1). The association between preconception diet quality and child BMI categories was not modified by pre-pregnancy BMI before and after adjusting for potential confounders.

Further sensitivity analysis showed that maternal diet quality was reasonably stable from before pregnancy to during pregnancy (Supplementary Table S2). There was a slight mean increase, 1.3 points SD (12.3) in the HEI-2015 score between before and during pregnancy, however, the mean change was not statistically significant (*p* = 0.07).

## 4. Discussion

This study was designed to investigate the relationship between maternal diet quality before conception and childhood BMI outcomes. We found that higher adherence to maternal diet quality before pregnancy was associated with a reduced risk of offspring being obese and underweight.

To our knowledge, this is the first evidence conducted on the association between preconception diet quality and offspring BMI outcomes in Australia. Our finding is contrary to that of Strohmaier et al. (2020) who found no relationship between healthful dietary intake during peri-conception and offspring obesity aged 12–23 years [19]. This inconsistency might be due to the difference in quality and quantity of maternal diets, the timing of the intake, offspring age, and the assessment techniques used. For example, Strohmaier et al. used Alternate Healthy Eating Index, (AHEI), Alternate Mediterranean Diet, and Dietary Approach to Stop Hypertension, to assess maternal diet quality. The offspring BMI was also categorized according to the International Obesity Task Force and WHO guidelines.

In reviewing the literature, several pieces of evidence have been documented in the relationship between women’s dietary consumption in pregnancy and childhood obesity [35,36,37]. However, no evidence was reported in the relationship between women’s diet quality and childhood underweight. Martin et al. observed a positive association between dietary patterns in pregnancy, characterized by white bread, processed and red meats, and French fries and offspring BMI-for-age z score in the first 3 years of life [35]. In a cross-generational cohort study conducted in Ireland, maternal intake of processed diets, characterized by chips, crisps, sweets, and chocolate, and processed meat was also significantly associated with increased risk of offspring overweight/obesity at age of 5 years [36]. On the other hand, Fernández-Barrés et al. reported higher adherence to Mediterranean dietary patterns, characterized by fruits, vegetables, legumes, nuts, cereals, olive oil, fish, dairy, and meat was associated with decreased risk of offspring abdominal obesity [37]. Chen et al. also reported that maternal dietary patterns, characterized by higher consumptions of vegetables and fruits and lower consumption of fast diet were associated with lowering the risk of child adiposity [38]. A Spanish birth cohort study reported a null association between Mediterranean diets and child BMI-Z score [37]. Dhana et al. also reported no association between women’s dietary quality explored by AHEI-2010 score and risk of childhood obesity [39]. In summary, there were inconsistent findings observed results in the assessment of maternal diets and offspring BMI. The discrepancies might be due to a large variation of women’s dietary consumptions, including the quantity and quality of diets. There was also variation in sample size, study design, and dietary assessment technique across the studies.

Maternal nutrition may affect early epigenetic changes and establishment of the gut microbiome in the fetus, which results in altered gene expression on adipogenesis leading to obesity and metabolic disease in offspring [9]. For example, in an animal study, Aagaard-Tillery et al. showed that maternal high-fat diets could change fetal chromatin structure and subsequent to increased recruitment of transcription factors to the target DNA binding sites through altering histone modification [40]. The high-fat diets could also affect fetal gut microbiome profiles, which contributes to the risk of offspring obesity [41]. Many studies showed that certain bioactive dietary components have a crucial role in influencing epigenetic modulation, known as “epigenetic diets”, including methyl donors (e.g., vitamin B12 and folate) source of diets, soybean isoflavone, broccoli sprouts, and green tea polyphenols [42,43]. Pre-pregnancy is the most critical period for epigenetic modulation since the immediate maturation of sperm and egg occurs during this period [44]. We, therefore, hypothesized that preconception diet quality would have a substantial influence on offspring weight outcomes.

Pre-pregnancy diet quality plays a vital role in proper fetal growth and development because the first trimester of gestation is a critical period for fetal and placental tissue developments [45]. A greater HEI-2015 score indicates a higher quality of dietary consumptions, including anti-inflammatory nutrients or antioxidants, unsaturated fats, and dietary fibers, and lower consumptions of saturated fats, sodium, refined grain, and added sugars. In the current study, the HEI-2015 score had also a strong inverse correlation with glycemic load, glycemic index, all fats, and saturated fats. Such a healthy dietary pattern might, therefore, have a beneficial role in childhood healthy weight outcomes.

Another important finding was that higher adherence to sodium intake was strongly linked with lowering the risk of childhood overweight and obesity, in which mothers with lower sodium consumption received greater scores. So far, no studies provide clear evidence that higher adherence to maternal sodium intake reduces the risk of childhood overweight/obesity. However, much evidence has shown that a greater sodium intake is linked with an increased risk of overweight/obesity [46,47]. A diet high in sodium enhances alteration of insulin and glucose metabolism which favors fat accumulation, subsequently increases adipose tissue mass [48]. Sodium intake also induces appetite and thirst and rises extracellular volume and energy intake, which contribute to overweight/obesity [49,50]. We have also observed that greater adherence to seafood and plant protein components was linked with a reduced risk of childhood underweight. Seafood and plant proteins are rich in high-quality proteins, n-3 polyunsaturated fatty acids, fiber, and essential micronutrients. These nutrients have a vital role in child growth and development, and healthy bodily compositions [51,52]. This finding is an important area for future research.

In the present study, the relationship between pre-pregnancy diet quality and offspring obesity was attenuated by pre-pregnancy BMI. Stuebe et al. [34] documented that pre-pregnancy BMI was a strong risk factor of offspring obesity. In our study, the pre-pregnancy BMI was also strongly linked with a child’s obesity in offspring. However, the association between preconception diet quality and childhood obesity did not vary by pre-pregnancy BMI. Further studies, therefore, could usefully explore the mediating effects of pre-pregnancy BMI in the relationship between preconception diet quality and offspring obesity.

This study has several strengths, including using a population-based prospective cohort study, a large sample size, and information on a wide range of potential confounders and covariates. The study used the HEI-2015 score to measure the pre-pregnancy dietary quality, which is a contemporary index of dietary quality where each component is calculated based on a density of 1000 Kcal. The HEI-2015 score assesses dietary quality rather than quantity. A validated and semi-quantitative FFQ was used to explore women’s dietary consumptions, which was designed for use in the Australian community. Further sensitivity analysis was performed to examine the stability and changes in women’s dietary quality from before to during pregnancy. In the current study, the HEI-2015 score was stable from before to during pregnancy, and no significant mean differences in the scores were observed at the two-time points. The study utilized data from a nationally representative cohort study of Australian women and children. The current findings, therefore, could be applicable to other populations. However, the present study was limited by the use of self-report data on women’s diets and offspring weight and height, which might have information bias and measurement error. The 101 food items were constructed according to women’s reports of dietary consumption over the previous 12 months, which might create a recall bias. Another limitation might be a long-time period between the outcome (offspring BMI between ages 2 and 12) and exposure (maternal diets). Some exposures/events might be occurred in this time window to influence offspring weight outcomes. Though this study controlled several potential confounders, there might be residual confounding, for example, micronutrient supplementation, which might alter the results.

## 5. Conclusions

In conclusion, the risk of childhood underweight and obesity were significantly decreased for those who had greater adherence to quality diet before pregnancy. The maternal dietary pattern is a potentially modifiable risk factor, and our findings highlight the importance of pre-pregnancy diet quality to enhance childhood health and quality of life. Further research in other populations is an essential next step in confirming the findings. This study also underscores the need for future well-powered longitudinal studies with adequate follow-up through childhood, adolescence, and adulthood in a specific age group to examine whether our results persist in later adulthood life.

## Figures and Tables

**Table 1:** Characteristics of the ALSWH mothers and their children according to offspring BMI category (*n* = 3391) ^a^.

Characteristics	All Children	Offspring BMI Category				*p-*Value ^b^
	*n* = 3391	Underweight	Normal	Overweight	Obese	
HEI-2015 score (%) of mothers						0.01
Quartile 1	25	13.9	69.3	12.3	4.5	
Quartile 2	25	12.5	71.2	13	3.3	
Quartile 3	25	9.9	74.1	12.6	3.4	
Quartile 4	25	9.8	76.1	12.2	1.9	
Maternal age (years), mean (SD)	33.1 (2.9)	32.9 (2.9)	33.1 (2.9)	33.2 (3.1)	33.2 (2.8)	0.41
Area of residence (%) ^c^						0.24
Urban	61.4	10.6	73.5	12.7	3.2	
Rural/remote	37.4	12.8	71.3	12.4	3.5	
Marital status (%)						0.52
Married	55.3	11.4	72.9	12.1	3.6	
De facto/separated/divorced	26.3	12.1	73.2	12.3	2.3	
Single	18.3	10.9	71.3	14	3.7	
Educational status (%) ^c^						0.002
Up to year 12 or equivalent	15.5	11.9	68.9	14.2	4.9	
Trade/apprenticeship/certificate/diploma	20.3	12.1	69.3	13.9	4.6	
University/higher degree	63.7	11.3	74.7	11.6	2.4	
Smoking status (%)						0.02
Never smoked	65.1	11.2	74.3	11.8	2.6	
Ex-smoker	19.5	11.9	70.4	13	4.7	
Current smoker	15.3	12.3	68.6	14.8	4.2	
Alcohol intake (%)						0.7
Non-drinker	6.2	11.5	71.8	13.4	3.3	
Rarely-drinker	19.9	10.6	71.7	13.3	4.3	
Low-risk drinker	70.6	11.7	73.1	12.2	2.9	
Risky-drinker	3.3	12.6	70.3	11.7	5.4	
Physical activity (%) ^c^						0.09
Sedentary/low, <600 MET min/week	39.4	12	70.6	13.8	3.7	
Moderate, 600 to 1200 MET min/week	26.4	11.7	75.1	11.2	2	
High, ≥1200 MET min/week	33.6	11	73.3	12.2	3.7	
Pre-pregnancy BMI (%) ^c^						<0.0001
Healthy weight, < 25 kg/m^2^	67.7	13	74.8	10.4	1.7	
Overweight, 25–30 kg/m^2^	21.5	9.1	71.7	15	4.3	
Obese, ≥30 kg/m^2^	10.5	7.3	61.1	20.5	11.2	
Total energy intake (KJ/day), mean (SD)	6752.9 (2238.3)	6799.8 (2209.1)	6702.3 (2215.3)	6826.5 (2334.5)	7430.9 (2378.8)	0.007
Household income (weekly) (%) ^c^						0.009
≤ 999$	16.5	13.1	69.3	12.2	5.4	
1000$–1499$	19.7	11.8	71.3	12.7	4.2	
≥1500$	51	10.9	75	11.8	2.3	
Don’t know/don’t want to answer	6.2	14.3	68.4	12.9	4.3	
Living alone	4.9	9.7	70.3	17.6	2.4	
Gestational diabetes mellitus (%) ^c^						0.33
Yes	4.6	15.4	69.2	10.9	4.5	
No	95	11.4	72.9	12.5	3.2	
Hypertensive disorder in pregnancy (%) ^c^						0.4
Yes	5.4	11.4	70.6	12.5	5.4	
No	94.2	11.5	72.9	12.5	3.1	
Preterm birth (%) ^c^						0.32
Yes	4.5	12.3	66.9	16.2	4.5	
No	95.2	11.5	73	12.3	3.2	
LBW (%) ^c^						0.05
Yes	3.2	18.3	69.7	7.3	4.6	
No	96.6	11.3	72.8	12.7	3.2	
Child age (years), mean (SD)	7.3 (2.9)	7.6 (2.9)	7.3 (2.8)	7.3 (3.1)	7.4 (2.9)	0.16
Child sex (%)						0.27
Male	53	10.9	74.1	11.9	3.1	
Female	47	12.2	71.1	13.2	3.5	
Breastfeeding status (%) ^c^						0.04
Never received	3.6	13.2	62.8	17.4	6.6	
Received	94.6	11.5	73	12.2	3.2	
Child diets ^c^, mean (SD)						
Fruits and vegetables	12.0 (3.6)	12.0 (3.7)	12.0 (3.6)	11.7 (3.6)	11.8 (4.4)	0.37
Sweetened beverages	0.6 (0.8)	0.6 (0.8)	0.5 (0.7)	0.5 (0.7)	0.8 (1.0)	0.001
Fat from dairy	3.0 (2.0)	3.0 (1.9)	3.0 (1.9)	3.1 (2.0)	3.1 (2.6)	0.84
Non-core foods	2.3 (1.0)	2.3 (1.0)	2.3 (1.0)	2.3 (0.9)	2.3 (1.1)	0.64

^a^ Values are mean (SD) or %, ^b^ *p-*values from ANOVA or Pearson chi-square, ^c^ missing values (Residence: *n* = 38, educational status: *n* = 16, physical activity: *n* = 18, Australian Longitudinal Study on Women’s Health (ALSWH), pre-pregnancy body mass index (BMI): *n* = 9, household income: *n* = 62, hypertensive disorder in pregnancy: *n* = 12, gestational diabetes mellitus: *n* = 14, preterm birth: *n* = 10, LBW: *n* = 5, breast feeding status: *n* = 63, fruits and vegetables: *n* = 64, fat from dairy: *n* = 108, sweetened beverages: *n* = 31, non-core food: *n* = 87).

**Table 2:** Maternal characteristics according to HEI-2015 score (*n* = 1936 mothers) ^a^.

	HEI-2015 Score				
Characteristics	Quartile 1	Quartile 2	Quartile 3	Quartile 4	*p-*Value ^b^
	**(*n* = 484)**	**(*n* = 484)**	**(*n* = 484)**	**(*n* = 484)**	
Maternal age (years), mean (SD)	31.6 (2.8)	32.4 (3.1)	32.2 (2.9)	32.7 (2.9)	<0.0001
Area of residence (%)					<0.0001
Urban	21.8	24.8	26.9	26.5	
Rural/remote	30.7	25.3	22	22	
Marital status (%)					0.2
Married	26.4	26.2	24.5	22.9	
De facto/separated/divorced	24.1	25	25.4	25.4	
Single	22.9	22.2	25.7	29.2	
Educational status (%)					<0.0001
Up to year 12 or equivalent	40.1	22.2	21.6	16.2	
Trade/apprenticeship/certificate/diploma	31.1	24.3	22.6	22.1	
University/higher degree	18.7	26.1	26.9	28.3	
Smoking (%)					<0.0001
Never smoked	23.6	25.1	27	24.4	
Ex-smoker	21.4	24.7	25.2	28.8	
Current smoker	34.2	25	17.6	23.2	
Alcohol intake (%)					0.001
Non-drinker	34.3	28.6	24.8	12.4	
Rarely-drinker	30.7	26.6	22.7	20	
Low-risk drinker	22.7	24.2	25.5	27.6	
Risky-drinker	25	26.5	27.9	20.6	
Physical activity (%)					<0.0001
Sedentary/low, <600 MET min/week	31.7	26	22.7	19.7	
Moderate, 600 to 1200 MET min/week	21.7	26.6	27.1	24.6	
High, ≥1200 MET min/week	19.9	22.7	25.9	31.5	
Pre-pregnancy BMI (%)					0.08
Healthy weight, < 25 kg/m^2^	24.6	24.5	24.6	26.3	
Overweight, 25–30 kg/m^2^	23.1	24.6	28.5	23.8	
Obese, ≥30 kg/m^2^	30.5	28.6	21.1	19.7	
Total energy intake (KJ/day), mean (SD)	7727.9 (2540.9)	68.94.2 (2143.6)	6247.5 (1880.3)	5912.5 (1842.5)	<0.0001
Household income (weekly) (%)					<0.0001
≤ 999$	37.5	27.8	17.9	16.8	
1000$–1499$	28	27.5	25.1	19.4	
≥1500$	20.3	23.6	26.6	29.5	
Don’t know/don’t want to answer	21.4	23	27.8	27.8	
Living alone	21.7	22.5	28.3	27.5	

^a^ Values are mean (SD) or %, ^b^ *p-*values from ANOVA or Pearson chi-square. Pre-pregnancy body mass index (BMI).

**Table 3:** Associations between offspring BMI category and pre-pregnancy HEI-2015 score (*n* = 3391 from 1936 mothers) ^a^.

Offspring BMI Category	HEI-2015 Score			
	Quartile 1	Quartile 2	Quartile 3	Quartile 4
Underweight				
Model 1	1	0.87 (0.64, 1.19)	0.66 (0.48, 0.92)	0.64 (0.46, 0.89)
Model 2 ^b^	1	0.89 (0.65, 1.21)	0.71 (0.50, 0.99)	0.64 (0.46, 0.90)
Model 3 ^c^	1	0.93 (0.67, 1.28)	0.76 (0.55, 1.07)	0.69 (0.49, 0.97)
Model 4 ^d^	1	0.94 (0.68, 1.29)	0.77 (0.55, 1.08)	0.68 (0.49, 0.96)
Normal ^e^	1	1	1	1
Overweight				
Model 1	1	1.03 (0.76, 1.39)	0.96 (0.70, 1.31)	0.90 (0.66, 1.24)
Model 2 ^b^	1	1.09 (0.80, 1.49)	1.04 (0.75, 1.43)	0.99 (0.70, 1.38)
Model 3 ^c^	1	1.15 (0.84, 1.59)	1.05 (0.75, 1.47)	0.98 (0.69, 1.38)
Model 4 ^d^	1	1.17 (0.85, 1.62)	1.08 (0.77, 1.52)	1.03 (0.72, 1.47)
Obese				
Model 1	1	0.72 (0.42, 1.23)	0.71 (0.42, 1.21)	0.38 (0.20, 0.74)
Model 2 ^b^	1	0.89 (0.51, 1.56)	0.97 (0.56, 1.67)	0.47 (0.24, 0.93)
Model 3 ^c^	1	0.98 (0.56, 1.72)	1.10 (0.64, 1.91)	0.49 (0.24, 0.98)
Model 4 ^d^	1	0.99 (0.56, 1.76)	1.12 (0.64, 1.97)	0.54 (0.26, 1.11)

Model 1 was crude RRR (95% CI). Model 2 was adjusted for maternal socio-demographic and lifestyle factors. Model 3 was adjusted as for model 2 and child characteristics. Model 4 was adjusted as for model 3 and pre-pregnancy BMI. Offspring body mass index (BMI). ^a^ Sample size refers to child-mother pairs (a mother could pair with up to 3 children). ^b^ Adjusted for women’s education, smoking status, physical activity, and income. ^c^ Adjusted for maternal education, smoking status, physical activity, income, child diets, sex, age, and breastfeeding status. ^d^ Adjusted for women’s education, smoking, physical activity, income, child diets, age, sex, breastfeeding, and pre-pregnancy BMI. ^e^ Reference group.

**Table 4:** Relationship between each dietary component of HEI-2015 scores and childhood BMI categories (*n* = 3391).

Components	Standard for Maximum Score	Standard for Minimum Score of Zero	Maximum Points	Offspring BMI Categories	Unadjusted RRR (95% CI) ^a^	Adjusted RRR (95% CI) ^b^
**Adequacy:**						
Total Fruits	≥0.8 C eq./1,000 kcal	No fruit	5	Underweight	0.99 (0.90, 1.09)	1.06 (0.95, 1.18)
				Overweight	0.90 (0.83, 0.99)	0.92 (0.83, 1.01)
				Obese	0.93 (0.78, 1.10)	1.13 (0.93, 1.37)
Whole Fruits	≥0.4 C	No whole fruit	5	Underweight	0.98 (0.88, 1.09)	1.06 (0.93, 1.21)
	eq./1000 kcal			Overweight	0.94 (0.85, 1.03)	0.99 (0.89, 1.11)
				Obese	0.89 (0.74, 1.06)	1.14 (0.90, 1.43)
Total Vegetables	≥1.1 C eq./1000 kcal	No vegetables	5	Underweight	0.98 (0.89, 1.07)	1.05 (0.94, 1.17)
				Overweight	0.96 (0.87, 1.05)	0.97 (0.87, 1.07)
				Obese	0.93 (0.80, 1.09)	1.07 (0.87, 1.30)
Greens and Beans	≥0.2 C eq./1000 kcal	No Greens and Beans	5	Underweight	1.10 (0.93, 1.30)	1.19 (0.99, 1.44)
				Overweight	0.95 (0.81, 1.10)	1.02 (0.85, 1.22)
				Obese	1.05 (0.79, 1.39)	1.36 (0.96, 1.94)
Whole Grains	≥1.5 oz. eq./1000 kcal	No whole grains	10	Underweight	0.97 (0.94, 0.99)	0.98 (0.94, 1.01)
				Overweight	1.00 (0.98, 1.03)	1.02 (0.98, 1.05)
				Obese	0.94 (0.89, 0.99)	0.97 (0.91, 1.03)
Dairy	≥1.3 C eq./1000 kcal	No dairy	10	Underweight	0.97 (0.94, 1.01)	1.03 (0.99, 1.08)
				Overweight	0.99 (0.96, 1.02)	1.00 (0.95, 1.05)
				Obese	0.94 (0.89, 1.00)	0.99 (0.92, 1.09)
Total Protein	≥2.5 oz. eq./1000 kcal	No protein foods	5	Underweight	0.82 (0.67, 1.01)	0.86 (0.69, 1.07)
				Overweight	0.96 (0.74, 1.24)	0.93 (0.71, 1.22)
				Obese	0.89 (0.65, 1.20)	0.91 (0.64, 1.29)
Seafood and Plant Proteins	≥0.8 C eq./1000 kcal	No seafood or plant proteins	5	Underweight	0.85 (0.74, 0.97)	0.84 (0.73, 0.99)
				Overweight	0.99 (0.89, 1.10)	1.03 (0.91, 1.18)
				Obese	0.99 (0.80, 1.22)	1.14 (0.89, 1.47)
Fatty Acids	(PUFA + MUFA)/SFA (g/day) ≥ 2.5	(PUFA + MUFA)/SFA (g/day) ≤1.2	10	Underweight	0.97 (0.94, 1.01)	0.98 (0.94, 1.02)
				Overweight	1.00 (0.97, 1.04)	1.01 (0.97, 1.05)
				Obese	0.96 (0.90, 1.03)	0.98 (0.91, 1.05)
**Moderation:**						
Refined Grains	≤1.8 oz. eq./1000 kcal	≥4.3 oz. eq./1000 kcal	10	Underweight	0.97 (0.94, 1.00)	0.99 (0.95, 1.03)
				Overweight	0.99 (0.96, 1.03)	0.99 (0.95, 1.03)
				Obese	0.99 (0.93, 1.06)	0.99 (0.93, 1.07)
Sodium	≤1.1 g/1000 kcal	≥2.0 g/1000 kcal	10	Underweight	1.05 (0.87, 1.26)	1.08 (0.89, 1.32)
				Overweight	0.96 (0.78, 1.20)	0.18 (0.14, 0.23)
				Obese	0.23 (0.18, 0.30)	0.21 (0.17, 0.26)
Added Sugars	≤6.5% of energy	≥26% of energy	10	Underweight	0.97 (0.90, 1.04)	1.00 (0.93, 1.08)
				Overweight	0.98 (0.91, 1.07)	0.99 (0.91, 1.08)
				Obese	0.93 (0.83, 1.04)	0.96 (0.85, 1.07)
Saturated Fats	≤8% of energy	≥16% of energy	10	Underweight	0.97 (0.95, 1.00)	1.00 (0.97, 1.05)
				Overweight	0.99 (0.96, 1.01)	0.99 (0.96, 1.03)
				Obese	0.94 (0.90, 0.99)	0.99 (0.93, 1.07)

Cup (C), ounce (oz.), monounsaturated fatty acids (MUFAs), polyunsaturated fatty acids (PUFAs), saturated fatty acids (SFAs). ^a^ Unadjusted model was crude RRR (95% CI). ^b^ Adjusted model was fully adjusted for maternal and child characteristics (women’s education, smoking, physical activity, income, child diets, sex, age, breastfeeding, total energy intake, pre-pregnancy BMI, and total HEI-2015 score).

## Data Availability

Data described in the manuscript, codebook, and analytic code will be made available upon request pending application and approval.

## Supplementary Materials

The following are available online at https://www.mdpi.com/2072-6643/13/4/1044/s1. Supplementary Table S1. Multinomial logistic regression model of offspring BMI categories with HEI-2015 score and pre-pregnancy BMI, with stratified interaction model. Supplementary Table S2. Stability and changes in HEI-2015 scores from before pregnancy to during pregnancy (*n* = 310) ^a^.

## Abbreviations

ALSWH	Australian Longitudinal Study on Women’s Health
ANOVA	analysis of variance
BMI	body mass index
CDQ	children’s dietary questionnaire
FFQ	food frequency questionnaire
GDM	gestational diabetes mellitus
HDP	hypertensive disorder in pregnancy
HEI-2015	healthy eating index
LBW	low birth weight
MET	metabolic equivalent
MatCH	Mothers and their Children’s Health
RRR	relative risk ratio
TEI	total energy intake
