# Supplementary material for: Pre-Pregnancy Diet Quality Is Associated with Lowering the Risk of Offspring Obesity and Underweight: Finding from a Prospective Cohort Study
Source: Nutrients. 2021 Mar 24;13(4):1044. doi: 10.3390/nu13041044 (PMC8063840; doi:10.3390/nu13041044)
Supplement: Supplementary file 1 [file nutrients-13-01044-s001.pdf]

Pre-pregnancy diet quality is associated with lowering risk of offspring obesity and underweight: finding from prospective cohort study

Dereje G. Gete

Online Supplementary Materials

Supplementary Table 1. Multinomial logistic regression model of offspring BMI categories with HEI-2015 score and pre-pregnancy BMI, with stratified interaction model

Offspring BMI categories	Pre-pregnancy BMI	HEI-2015 score	Unadjusted RRR (95% CI)	Adjusted RRR (95% CI) [a]
Underweight	Healthy weight, < 25 kg/m^2	Quartile 1	1.00	1.00
		Quartile 2	0.84 (0.58, 1.21)	0.89 (0.61, 1.30)
		Quartile 3	0.71 (0.49, 1.02)	0.84 (0.57, 1.23)
		Quartile 4	0.73 (0.50, 1.05)	0.79 (0.54, 1.15)
	Overweight, 25 - <30 kg/m^2	Quartile 1	1.00	1.00
		Quartile 2	1.04 (0.48, 2.21)	1.06 (0.47, 2.39)
		Quartile 3	0.74 (0.32, 1.72)	0.68 (0.28, 1.65)
		Quartile 4	0.56 (0.24, 1.34)	0.56 (0.22, 1.41)
	Obese, ≥30 kg/m^2	Quartile 1	1.00	1.00
		Quartile 2	1.46 (0.48, 4.40)	1.52 (0.49, 4.68)
		Quartile 3	0.80 (0.20, 3.09)	0.76 (0.19, 2.98)
		Quartile 4	0.33 (0.06, 1.74)	0.35 (0.06, 1.87)
	p-values for interaction		0.48	0.46
Overweight	Healthy weight, < 25 kg/m^2	Quartile 1	1.00	1.00
		Quartile 2	1.25 (0.82, 1.89)	1.42 (0.91, 2.20)
		Quartile 3	1.08 (0.70, 1.66)	1.18 (0.75, 1.85)
		Quartile 4	1.21 (0.80, 1.84)	1.29 (0.82, 2.03)
	Overweight, 25 - <30 kg/m^2	Quartile 1	1.00	1.00
		Quartile 2	0.70 (0.33, 1.46)	0.65 (0.30, 1.43)
		Quartile 3	0.91 (0.44, 1.91)	0.91 (0.41, 1.98)
		Quartile 4	0.55 (0.26, 1.18)	0.49 (0.22, 1.11)
	Obese, ≥30 kg/m^2	Quartile 1	1.00	1.00
		Quartile 2	0.75 (0.31, 1.78)	0.67 (0.27, 1.66)
		Quartile 3	0.78 (0.34, 1.82)	0.63 (0.24, 1.65)
		Quartile 4	0.56 (0.22, 1.43)	0.65 (0.25, 1.69)

	p-values for interaction		0.71	0.58
Obese	Healthy weight, < 25 kg/m^2	Quartile 1	1.00	1.00
		Quartile 2	0.42 (0.16, 1.09)	0.65 (0.24, 1.77)
		Quartile 3	0.80 (0.37, 1.74)	1.33 (0.57, 3.10)
		Quartile 4	0.51 (0.20, 1.31)	0.74 (0.28, 1.96)
	Overweight, 25 - <30 kg/m^2	Quartile 1	1.00	1.00
		Quartile 2	2.35 (0.51, 10.85)	1.76 (0.40, 7.69)
		Quartile 3	0.98 (0.24, 4.03)	0.75 (0.19, 2.88)
		Quartile 4	1.27 (0.25, 6.45)	0.99 (0.19, 5.05)
	Obese, ≥30 kg/m^2	Quartile 1	1.00	1.00
		Quartile 2	2.39 (0.64, 8.84)	1.85 (0.46, 7.49)
		Quartile 3	0.99 (0.28, 3.47)	0.70 (0.19, 2.60)
		Quartile 4	0.29 (0.05, 1.77)	0.14 (0.01, 1.42)
	p-values for interaction		0.47	0.51

[a] Adjusted for maternal education, smoking status, physical activity, household income, child diets, sex, age, and breast feeding status. In both models, women with a lowest quartile of HEI-2015 score and children with normal weight are used as reference groups.

S**upplementary table 2.** Stability and changes in HEI-2015 scores from before pregnancy to during pregnancy (n= 310) [a]

Dietary patterns	Period	Mean (SD)	Spearman's correlation coefficient (r)
HEI-2015 score	Before pregnancy	58.4 (12.1)	0.38
	During pregnancy	59.7 (10.9)	
	Mean difference	1.3 (12.7)	
	P-value	0.07	< 0.0001

[a] Values are mean (SD) and correlation coefficients (r), p-values from and paired t-test and Spearman's correlation
